# Spontaneous assembly of chemically encoded two-dimensional coacervate droplet arrays by acoustic wave patterning

**DOI:** 10.1038/ncomms13068

**Published:** 2016-10-06

**Authors:** Liangfei Tian, Nicolas Martin, Philip G. Bassindale, Avinash J. Patil, Mei Li, Adrian Barnes, Bruce W. Drinkwater, Stephen Mann

**Affiliations:** 1 Centre for Protolife Research and Centre for Organized Matter Chemistry, School of Chemistry, University of Bristol, Bristol BS8 1TS, UK; 2 Faculty of Engineering, Queens Building, University of Bristol, Bristol BS8 1TR, UK; 3 School of Physics, HH Wills Physics Laboratory, University of Bristol, Bristol BS8 1TL, UK

## Abstract

The spontaneous assembly of chemically encoded, molecularly crowded, water-rich micro-droplets into periodic defect-free two-dimensional arrays is achieved in aqueous media by a combination of an acoustic standing wave pressure field and *in situ* complex coacervation. Acoustically mediated coalescence of primary droplets generates single-droplet per node micro-arrays that exhibit variable surface-attachment properties, spontaneously uptake dyes, enzymes and particles, and display spatial and time-dependent fluorescence outputs when exposed to a reactant diffusion gradient. In addition, coacervate droplet arrays exhibiting dynamical behaviour and exchange of matter are prepared by inhibiting coalescence to produce acoustically trapped lattices of droplet clusters that display fast and reversible changes in shape and spatial configuration in direct response to modulations in the acoustic frequencies and fields. Our results offer a novel route to the design and construction of ‘water-in-water' micro-droplet arrays with controllable spatial organization, programmable signalling pathways and higher order collective behaviour.

## Abstract (web summary)

Isolated droplets can be used as micro-reactors, yet it is challenging to operate them functionally in solution and observe chemical exchanges between droplets. Here, Tian *et al*. use an acoustic trap to assemble water-based micro-droplets into periodic arrays, spontaneously separated from solution media.

## Graphical abstract

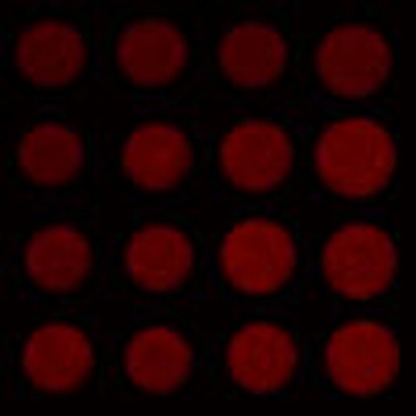

Miniaturization of fluid compartments in the form of liquid micro-droplets is important in diverse scientific areas^1^ such as chemical and biochemical analysis^2^,^3^, protein crystallization^4^ and micro-reactor technology^5^. Many of these applications require high-throughput analyses of spatially addressable arrays of liquid micro-droplets over a range of timescales and chemical/physical environments. Typically, arrays of droplets with a uniform size have been prepared by microfluidics^6^,^7^, microfabrication^8^,^9^,^10^, printing^11^,^12^ and by application of electrical^13^ or magnetic fields^14^. The droplets are stabilized by immersion in an appropriate continuous phase (water droplets in oil for example) or exposure on a dry surface, which lead to patterns of physically isolated droplets, which can then be exploited as independent micro-reactors that are essentially free from cross-contamination. On the other hand, isolation of the droplets within the arrays is not compatible with dynamical interactions such as triggering chemical signals between the droplets or enabling the droplets to communicate with and respond to time-dependent changes in their external environment. To achieve these dynamical interactions, new technologies are required that provide the production and organization of liquid micro-droplets with similar polarity to the associated continuous phase, such as the formation and patterning of water-rich droplets in a continuous aqueous phase. Such systems are characterized by a relatively low surface tension between the droplets and continuous phase, and remain technically challenging. In this regard, recent studies have described the formation of aqueous coacervate micro-droplets in a water continuous phase using a parallel-flow focusing microfluidic device^15^, and the printing of water-rich droplets in an aqueous phase using a dextran/polyethylene glycol (PEG)-based aqueous two-phase system^16^. However, the spontaneous organization of water-rich droplets in an aqueous phase into two-dimensional (2D) arrays with non-close packed lattices remains a major challenge.

In this paper, we demonstrate the spontaneous assembly and spatial organization of water-rich molecularly crowded micro-droplets to form 2D arrays in aqueous media. Droplet assembly is achieved by a spontaneous process of complex coacervation^17^,^18^,^19^, which is a liquid–liquid phase separation phenomenon driven by attractive electrostatic interactions usually between counter-charged polyelectrolytes, and entropic gains from the release of small, bound counter-ions and restructuring of water molecules. The resultant micron-sized coacervate droplets comprise a dense, component-enriched viscoelastic phase, dispersed in a chemically deficient aqueous continuous phase. Coacervate droplets have been used for storage of food additives^19^,^20^, drug delivery^21^,^22^, protein purification^23^, and more recently, exploited as membrane-free protocells^24^,^25^,^26^ capable of enhanced enzymatic activity^27^, electric field-induced energization^28^, and *in vitro* gene expression^29^. Herein, we show that the *in situ* generation of coacervate micro-droplets and their spatial organization into 2D periodic lattices in water can be achieved without direct contact by acoustic trapping methods. Acoustic radiation forces depend on the acoustic contrast generated by compositional differences between media^30^, and although acoustic beams and standing waves have been exploited for multi-dimensional trapping, patterning and manipulation of micron-sized particles, aqueous droplets in oil and intact cells^31^,^32^,^33^,^34^,^35^,^36^,^37^,^38^,^39^, generating defect-free uniform patterns with a single particle positioned at each acoustic pressure node has only been achieved at a highly specific ratio of particle size to acoustic standing wavelength^36^. Significantly, acoustic trapping has not been used to generate arrays of water-rich droplets dispersed in an aqueous medium, principally because of the low interfacial tension of the system.

Using an acoustic standing wave trap, we demonstrate the spontaneous assembly and organization of polydiallydimethylammonium chloride (PDDA)/adenosine 5^/^-triphosphate (ATP) coacervate micro-droplets into defect-free arrays with controllable lattice spacing and droplet size. We show that individual PDDA/ATP droplets of near uniform size, typically 50–100 μm in diameter, are produced in the acoustic field by *in situ* coalescence of sub-micrometer-sized droplets that aggregate specifically at the Gor'kov potential energy minima (acoustic pressure nodes) of the standing wave in the early stages of pattern formation. Significantly, coalescence between the primary droplets can be curtailed by adjusting the composition of the coacervate droplets such that localized aggregates of closely packed droplets are produced at each node in the acoustic pressure field. The localized clusters exhibit collective responses to modulations in the acoustic standing wave to produce arrays with reversible dynamical properties based on transformations in droplet shape and exchange of matter between adjacent nodes in the acoustic field.

Our methodology is applicable to a wide range of complex coacervate systems involving proteins, DNA, polysaccharides, nucleotides and synthetic polyelectrolytes. Coacervates exhibiting strong interactions with the underlying substrate remained spatially patterned when the acoustic field is switched off, while those showing reduced surface pinning produce arrays of single micro-droplets that display spatially confined dynamic motions such as localized spinning. Moreover, periodic arrays of chemically encoded single droplets containing sequestered dye molecules, proteins, enzymes, nanoparticles or microparticles can be readily produced *in situ* during droplet assembly or partitioned into the patterned arrays post-assembly. By adjusting the ratio of PDDA and ATP to limit local molecular diffusion we demonstrate that acoustically patterned populations of coacervate droplets containing different chemical information can be spatially positioned within the sample chamber of the trapping device. Finally, we show that it is possible to transit a reaction wavefront through an array of acoustically trapped enzyme-containing coacervate micro-droplets by establishing an appropriate chemical gradient within the sample chamber of the device.

## Results

### Acoustic patterning of coacervate micro-droplet arrays

**Figure 1:**
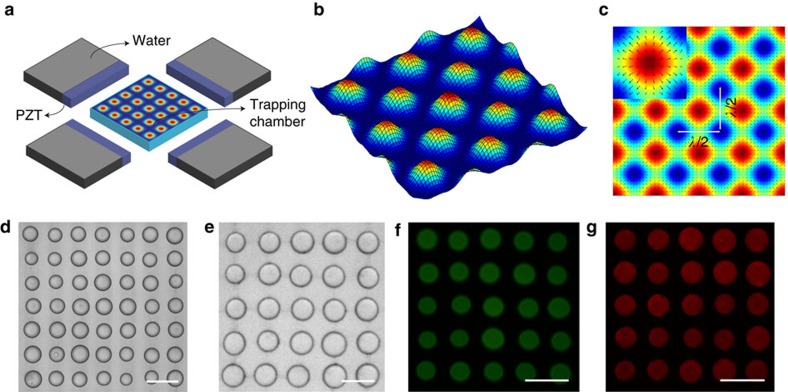
Acoustic patterning of coacervate micro-droplet arrays. (**a**) Schematic representation of the acoustic trapping device. Four piezoelectric transducer (PZT) elements (purple cuboids) are arranged around a central square sample chamber, and driven as two pairs. Coacervate micro-droplets are spontaneously assembled and patterned into 2D arrays within the chamber due to the periodic acoustic standing wave pressure field. Additional chambers behind each of the PZTs are filled with water to provide cooling. (**b**) Simulation of the acoustic pressure distribution in the acoustic trapping device; high pressure (red), low pressure (blue). Gradients in acoustic pressure cause the coacervate micro-droplets to be forced towards the acoustic pressure nodes (blue). (**c**) Simulation showing the Gor'kov potential distribution in the acoustic trapping device. The separation distance between the nodes is half of the acoustic wavelength (*λ*). Inset shows single anti-node with local directions of the acoustic radiation force (arrows). (**d**,**e**) Optical microscopy images of acoustically patterned PDDA/ATP droplets produced using transducer pairs operated at 6.76/6.78 MHz (10 V) by addition of ATP (100 μl, 50 mM) to PDDA (1 ml, 5 mM monomer, 100–200 kDa) contained in the sample holder (**d**), or at 4.99/5.00 MHz (10 V) (ATP, 200 μl, 50 mM; PDDA, 2 ml, 5 mM monomer) (**e**). The lattice spacing is increased at the lower acoustic frequency. Mean size of the droplets (*ca*. 110 μm) in **e** is larger because of the increased amounts of PDDA and ATP used in the preparations. (**f**,**g**) Fluorescence microscopy images of TNP-ATP (0.1 mol%) (**f**) and RITC-PAH-doped (10 mol%) (**g**) PDDA/ATP droplets showing the presence of TNP-ATP and PAH throughout the interior of the coacervate phase. Images shown in **d**,**f**,**g** and **e** were recorded at 45 and 30 min, respectively, after mixing the PDDA and ATP solutions. Scale bars, 150 μm.

The spontaneous assembly and patterning of molecularly crowded PDDA/ATP coacervate micro-droplets in water was undertaken using a custom-made acoustic trapping device that was fabricated from polyethylene terephthalate and consisted of a central square chamber and four piezoelectric transducers arranged around the periphery (Fig. 1a and Supplementary Fig. 1). Opposing transducers were wired in parallel and driven as a pair with a sinusoidal voltage supply by a signal generator. Each of the two orthogonally arranged pairs was operated at slightly different frequencies such that the total acoustic radiation force field tended to the sum of the force fields of the individual pairs at times greater than 8 μs (see Methods). As the response time of the droplets was considerably greater than 8 μs, this simple summation approach was valid for all the experiments undertaken. Simulations of the resultant acoustic standing wave pressure field showed a periodic array of nodes and anti-nodes in a 2D grid-like pattern that were associated with the minima (pressure nodes) and maxima (pressure antinodes), respectively, of the Gor'kov potential energy distribution (Fig. 1b,c). An aqueous solution of PDDA (*M*_w_=100–200 kDa) was placed into the central chamber and aqueous ATP then added in the presence of an acoustic standing wave field generated from orthogonally arranged opposing transducer pairs typically operating at 6.76/6.78 MHz or 4.99/5.00 MHz with corresponding wavelengths of 219/218 μm and 297/296 μm, respectively (see Methods). Vigorous mixing of the solutions gave rise to spontaneous liquid–liquid phase separation and the formation of molecularly crowded PDDA/ATP coacervate droplets that exhibited sufficient acoustic contrast to become trapped by the acoustic radiation force. Migration and trapping of the droplets at the pressure nodes of the acoustic field was consistent with the increased density and bulk modulus of the coacervate micro-droplets compared with the aqueous continuous phase. Moreover, coalescence of the trapped coacervate droplets in the pressure node was accelerated due to the second acoustic radiation force that acts over short distances^32^. As a consequence, the micro-droplets although initially trapped in the three-dimensional field, slowly sedimented under gravity onto the PEG functionalized glass substrate to produce a defect-free square array of uniform-sized droplets with a lattice spacing that was determined by half of the acoustic wavelength. Typically, centre-to-centre spacings of 110 and 150 μm were observed for arrays produced under acoustic frequencies of 6.76/6.78 and 4.99/5.00 MHz, respectively (Fig. 1d,e). Doping of the polymer/nucleotide mixtures with rhodamine isothiocyanate (RITC)-tagged polyallylamine hydrochloride (PAH; polycationic polymer) or trinitrophenol-ATP (TNP-ATP) produced acoustically ordered arrays of fluorescent micro-droplets (Fig. 1f,g), and confirmed the presence of the complex coacervate phase within the droplets.

**Figure 2:**
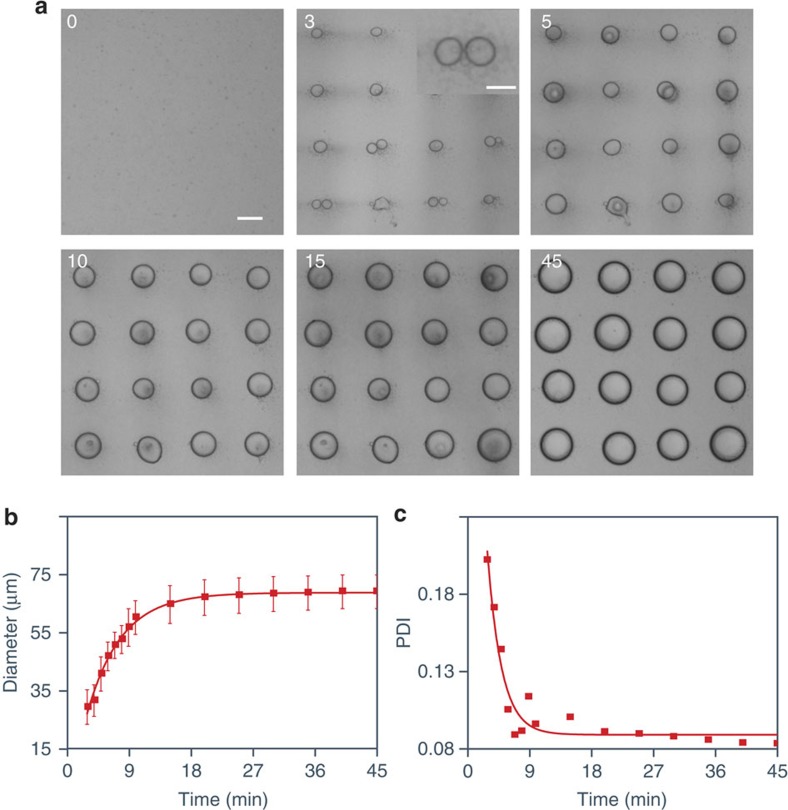
Growth of acoustically trapped coacervate micro-droplets. (**a**) Time-dependent optical microscopy images showing the growth of PDDA/ATP coacervate droplets at the nodes of an acoustic standing wave pressure field generated by transducer pairs operating at 6.76/6.78 MHz (10 V). The images were recorded at *t*=0, 3, 5, 10, 15 and 45 min after addition of ATP (100 μl, 50 mM) to a PDDA solution (1 ml, 5 mM monomer, 100–200 kDa) contained within the sample chamber of the acoustic trapping device. Inset at *t*=3 min: high-magnification image showing localized aggregate of coalescing primary droplets; sub-micrometre-sized droplets are observed as indistinct areas of higher optical contrast surrounding the two larger droplets undergoing coalescence; scale bars, 20 μm (inset) and 50 μm. (**b**) Plot showing change in mean diameter of PDDA/ATP coacervate micro-droplets against time during growth within the acoustic field under the above conditions. An induction time of *ca.* 3 min was observed during which the primary sub-micrometer-sized droplets sedimented onto the glass substrate. Error bars represent the standard deviation of the size of coacervate micro-droplets at different time intervals in the same device. (**c**) Corresponding time-dependent plot of the polydispersity index (PDI) for PDDA/ATP micro-droplets shown in **b** after mixing PDDA and ATP.

Optical microscopy video monitoring of the formation of single droplets within the acoustic trap operating at 6.76/6.78 MHz (10 V) indicated that they were produced simultaneously by *in situ* coalescence of sub-micrometer-sized primary droplets that accumulated around each node in the early stages of pattern formation (Fig. 2a and Supplementary Movie 1). Accumulation and coalescence of multiple primary droplets at each acoustic node was predominant up to 5 min after mixing PDDA (1 ml, 5 mM monomer, 100–200 kDa) and ATP (100 μl, 50 mM), after which the trapped single droplets grew slowly over a period of 45 min to attain a near uniform size with a mean diameter of approximately 70 μm. When all the nodes were filled with droplets (typically after 3 min), a plot of the average droplet diameter against time was fitted to an exponential function (*R*^2^=0.991) (Fig. 2b), and measurements of the droplet polydispersity index showed a marked increase in particle size homogeneity within the first 9 min of acoustic trapping (Fig. 2c). Thus, it was possible to control the size of the droplets while maintaining a constant lattice spacing by removing the supernatant from the sample chamber after various time intervals to quench the coalescence process. For this, we used the pseudo-kinetic plots shown in Fig. 2b as a calibration curve to guide the preparation of 2D arrays with fixed spacing and geometry but variable droplet size. Increasing the initial PDDA and ATP concentrations accelerated droplet growth for the same acoustic frequencies by increasing the rate of coalescence at the acoustic nodes. For example, increasing the PDDA and ATP concentrations to 7.5 and 75 mM, respectively, produced droplets with a larger mean diameter (83 μm) within a shorter time period (18 min) (Supplementary Fig. 2). Merging of single droplets located at adjacent positions in the 2D array was also more apparent at higher polymer concentrations (Supplementary Fig. 3). Conversely, decreasing the initial concentrations in the acoustic trapping device reduced the rate of droplet coalescence at the acoustic nodes (Supplementary Fig. 4). In general, the ratio (*R*_*D/λ*_ ) of droplet diameter (*D*) to wavelength (*λ*) was in the range 0<*R*_*D/λ*_<0.36, with an upper limit that was smaller than expected theoretically (0<*R*_*D/λ*_<0.5) due to premature merging of adjacent droplets trapped at the nodes by accumulation of relatively large droplets growing in the suspension during the patterning process.

Similar procedures were used to self-assemble, trap and acoustically pattern PDDA-containing coacervate micro-droplets prepared from a range of components including proteins (bovine serum albumin, BSA), polysaccharides (carboxymethyldextran, CM-D), polynucleotides (double stranded DNA (dsDNA)) and anionic polymers (polyacrylic acid, PAA), as well as coacervates formed by complexation of polyethylenimine (PEI) and CM-D. In general, the PDDA-containing coacervates were prepared by placing the above solutions into the sample chamber of the device followed by addition of PDDA (*M*_w_=8.5 kDa) in the presence of two orthogonal acoustic standing waves (6.76/6.78 MHz, 10 V) (see Methods). In each case, 2D arrays of spatially organized single micro-droplets could be produced in aqueous media via coalescence of primary droplets at the acoustic nodes (Supplementary Figs 5–9).

### Dynamical behaviour in coacervate micro-droplet arrays

Given the propensity for coacervates to adopt a range of physical and chemical properties depending on their composition, surface charge, dehydration and complexation strength^17^,^18^,^19^,^20^,^21^,^22^,^23^, we developed experimental procedures for controlling the dynamical behaviour of the acoustically patterned droplets when immersed in aqueous media. Binding of the droplets to the underlying PEGylated glass substrate was modulated by changes in composition and exploited to induce droplet rotation and provide a method for immobilizing or dispersing the arrays in water when the acoustic field was switched off. Acoustically trapped PDDA (100–200 kDa)/ATP single droplets were so strongly attached to the substrate that they adopted a hemispherical morphology and exhibited minimal rotational movement in the presence of the acoustic field (Supplementary Fig. 10). As a consequence, they remained spatially fixed at the lattice positions for extended periods of time (*t*≥12 h) when the acoustic pressure was switched off. Acoustically trapped PDDA (8.5 kDa)/CM-D single droplets in contrast were less firmly attached to the PEGylated glass substrate and displayed spatially confined localized spinning at the potential energy minima of the pressure field possibly due to an acoustic streaming force (Supplementary Movie 2). Attachment of the PDDA/CM-D droplets was further reduced by addition of high molecular weight fluorescein isothiocyanate (FITC)-CM-D (25 mol%) such that the arrays slowly dispersed into bulk solution when the acoustic field was removed.

**Figure 3:**
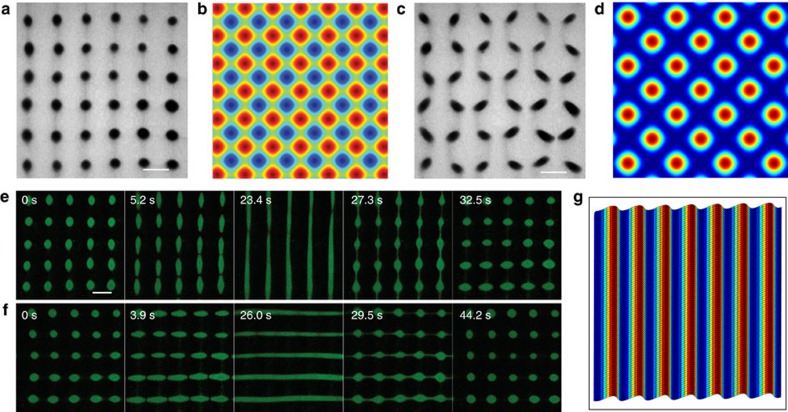
Dynamical behaviour of acoustically trapped coacervate micro-droplets. (**a**) Optical microscopy image showing acoustically patterned 2D array comprising discrete spherical aggregates of non-coalescing sub-micrometre-sized PDDA/FITC-CM-D/CM-D coacervate droplets. The optical contrast originates from the collection of micro-droplets within each aggregate; individual droplets are too small to be clearly resolved. The droplets were prepared by adding PDDA (100 μl, 50 mM monomer, 8.5 kDa) to 1 ml of a CM-D (36 mM)/FITC-CM-D (9 mM) mixture contained within the sample chamber of an acoustic trapping device using two orthogonal transducer pairs operating at different frequencies (6.76/6.78 MHz, 10 V); scale bar, 100 μm. (**b**) Simulation of the acoustic field shown in **a** showing the Gor'kov potential distribution in the acoustic trapping device; high pressure (red), low pressure (blue). (**c**) Optical microscopy image of sample shown in **a** but using orthogonal transducer pairs operating in-phase at the same frequency (6.76 MHz, 10 V) to produce a 2D array of non-spherical coacervate micro-droplet aggregates; scale bar, 100 μm. (**d**) Simulation of the acoustic field shown in **c** showing the Gor'kov potential distribution in the acoustic trapping device; high pressure (red), low pressure (blue). (**e**,**f**) Time-dependent series of fluorescence microscopy images showing acoustically induced reversible transformation of a square lattice of discrete spherical aggregates of non-coalescing sub-micrometre PDDA/FITC-CM-D/CM-D coacervate droplets into elliptical arrangements and continuous vertical fringes (**e**) followed by recapitulation of the 2D array and subsequent reversible manipulation of the square lattice into horizontal lines of non-interacting coacervate micro-droplets (**f**); *t*=time in seconds after switching on/off the corresponding pairs of piezoelectric transducers (PZTs); scale bar, 100 μm. (**g**) Simulation of the acoustic pressure distribution in the acoustic trapping device operating with only one pair of PZTs; high pressure (red), low pressure (blue).

Using a similar strategy, changes in composition, PDDA molecular weight or charge ratio between the coacervate components were exploited to promote or inhibit single droplet formation at the nodes of the acoustic standing wave pressure field by influencing the interfacial tension between the coalescing primary droplets. Whereas 2D arrays of polymer-protein PDDA/BSA single droplets were produced using a low molecular weight PDDA (8.5 kDa), replacing the polymer with a 100–200 kDa PDDA gave rise to a square grid comprising aggregates of non-coalescing PDDA/BSA droplets that remained trapped at the acoustic nodes (Supplementary Fig. 11). We attributed these differences to increases in the relaxation time and viscosity of the coacervate droplets prepared with high molecular weight PDDA (ref. 40). Aggregates of non-coalescing primary droplets with a mean diameter of 310 nm and net negative surface charge (−20 mV) (Supplementary Fig. 12) were also patterned in acoustically trapped arrays prepared from mixtures of PDDA (8.5 kDa), FITC-tagged CM-D and CM-D (Fig. 3a,b). Significantly, by using FITC-CMD in the above preparations, we were able to generate ordered arrays of aggregates that were only loosely attached to the underlying substrate. As a consequence, modulations in the acoustic radiation force could be used to generate fast (<1 min) and reversible changes in the shape and spatial configuration of the droplet aggregates to produce dynamical patterns capable of exchanging matter between adjacent nodes in the acoustic field. For example, by exciting transducer pairs that operated in-phase at the same frequency (6.76 MHz) we were able to transform the localized aggregates into new spatial configurations (Fig. 3c,d and Supplementary Movie 3)^41^. In addition, patterns of discrete spherical aggregates could be reversibly transformed in aqueous media into vertically or horizontally oriented elliptical shapes, further distorted into continuous fringes of micrometre-sized coacervate droplets, and then recapitulated by switching on/off the corresponding pairs of piezoelectric transducers (Fig. 3e–g and Supplementary Movie 4).

### Molecular uptake and spatially positioned enzyme activity

**Figure 4:**
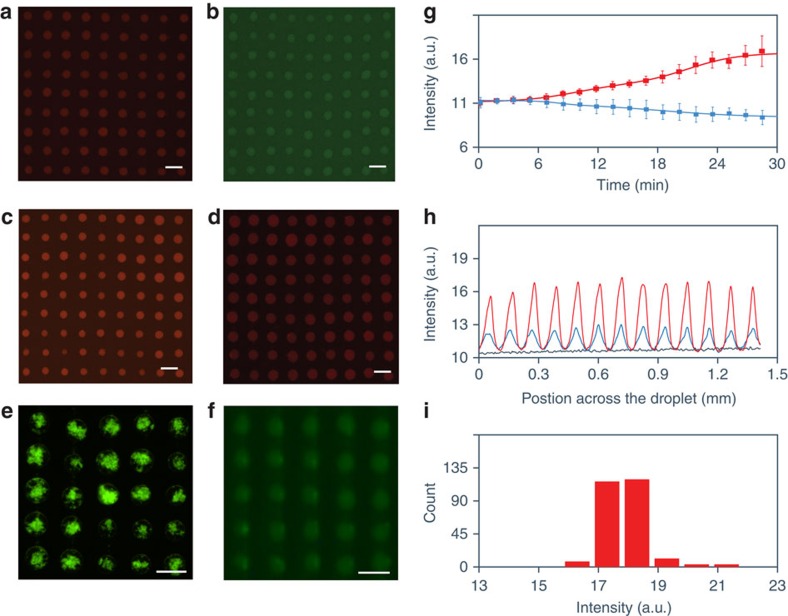
Chemical encoding of acoustically patterned coacervate micro-droplets. (**a**–**f**) Fluorescence microscopy images of acoustically patterned PDDA/ATP coacervate droplets containing methylene blue (**a**), calcein (**b**), sulforhodamine B (**c**), nile red (**d**), FITC-labelled 100 nm-sized polystyrene particles (partition constant=4,000) (**e**) or FITC-GOx (**f**); scale bars, 100 μm. (**g**) Plots of time-dependent increase and decrease in FITC-GOx mean fluorescence intensity measured at the acoustic nodes (red curve) and antinodes (blue curve), respectively, after addition of ATP (50 μl, 25 mM) to a premixed solution of PDDA (1 ml, 2.5 mM, monomer, 8.5 kDa) and FITC-GOx (1 μg ml^−1^) solution under an acoustic standing wave pressure field (6.76/6.78 MHz, 10 V). Error bars represent the standard deviation of the mean fluorescence intensities at acoustic nodes (red) or antinode (blue) at different time intervals in the same device. (**h**) Mean fluorescence line intensity profiles recorded across FITC-GOx-containing PDDA/ATP coacervate droplets prepared as in **g** after 0 (black line), 10 (blue line) and 30 min (red line) of exposure in the acoustic trapping field. (**i**) Statistical counts of mean fluorescence intensity of FITC-GOx-containing PDDA/ATP droplets after 30 min. Sample was prepared as in **g**. Images shown in **a**–**e** and **f** were recorded at 45 and 30 min, respectively, after mixing. a.u., arbitrary unit.

We prepared acoustically trapped arrays of chemically encoded single droplets by spontaneously sequestering water-soluble organic dyes of different charge, proteins, enzymes, polystyrene nanoparticles or silica microparticles into the molecularly crowded PDDA/ATP droplets during or after the assembly process in aqueous media (Fig. 4a–f and Supplementary Fig. 13). For example, uptake of FITC-tagged glucose oxidase (FITC-GOx) was achieved by injecting an ATP solution into a pre-mixed PDDA/FITC-GOx solution contained within the device under an acoustic radiation force field (Supplementary Fig. 14). Measurements of the time-dependent changes in FITC-GOx mean fluorescence intensity at the acoustic pressure nodes and antinodes showed progressive increases and decreases, respectively, over the initial 30 min period (Fig. 4g), indicating that FITC-GOx was spontaneously sequestered into the assembling coacervate droplets. Corresponding mean fluorescence line intensity measurements recorded over the initial 30 min across a row of FITC-GOx-containing PDDA/ATP coacervate droplets showed the emergence of a periodic one-dimensional profile with a spacing of *ca*. 110 μm (Fig. 4h) and highly uniform local intensity distributions (Fig. 4i), indicating that droplets with comparable enzyme concentrations could be readily produced by acoustic trapping.

**Figure 5:**
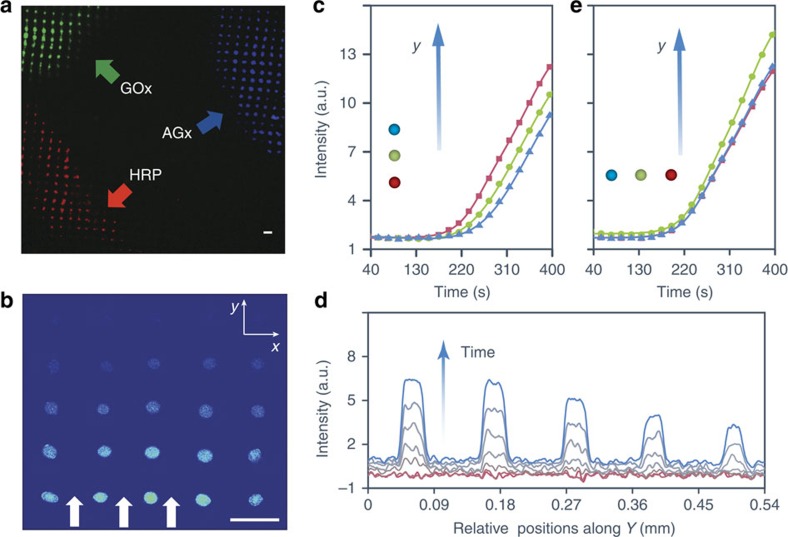
Spatial positioning of mixed populations and enzyme activity. (**a**) Fluorescence microscopy image showing three spatially positioned domains of enzyme-containing PDDA (100–200 kDa)/ATP droplet arrays with sequestered GOx (green), AGx (blue) or HRP (red) (see arrows). The ensemble was prepared by sequentially adding premixed solutions of PDDA (1 μl, 25 mM, monomer)/FITC-GOx (0.2 mg ml^−1^), PDDA (1 μl, 25 mM monomer)/AGx (0.2 mg ml^−1^), or PDDA (1 μl, 25 mM, monomer)/RITC-HRP (0.2 mg ml^−1^) to ATP (1 ml, 2.5 mM) contained within the acoustic trapping device operating under an acoustic standing wave pressure field (6.76/6.78 MHz, 10 V). (**b**) Fluorescence microscopy image of an acoustically patterned 2D array of 110 μm-spaced HRP-containing PDDA (100–200 kDa)/ATP coacervate droplets recorded 250 s after diffusion of H_2_O_2_/*o*-PD into one side of the reaction chamber along the *y* direction (arrows). Formation of the fluorescent 2,3-DAP product is observed in the form of a chemical wave-front that transits across the droplet array; scale bars in **a** and **b**, 100 μm. (**c**) Plots of time-dependent changes in 2,3-DAP fluorescence mean intensity associated with patterned HRP-containing PDDA/ATP coacervate micro-droplets positioned 110 μm apart along a single row aligned parallel to the direction of H_2_O_2_/*o*-PD diffusion (*y* axis in **b**). Plots for three droplets positioned at alternate lattice points lying parallel to the diffusion direction (*y* axis) and at increasing distances from the advancing wave-front (red<green<blue) are shown. A time lag of *ca*. 12 s is observed between droplets position on adjacent lattice points. (**d**) Fluorescence line intensity profiles recorded across a single row of HRP-containing PDDA/ATP droplets aligned along the direction of diffusion (*y* axis in **b**) 50, 100, 150, 200, 225, 250, 275 and 300 s after injection of H_2_O_2_/*o*-PD into the reaction chamber. (**e**) Similar plots as in **c** but for three droplets positioned at alternate lattice points in a row lying perpendicular to the diffusion front showing minimal differences in their enzymatic activity at a given time. a.u., arbitrary unit.

Based on the capability of the acoustically trapped coacervate droplets to sequester a range of water-soluble molecules during pattern formation, we developed a method for preparing spatially positioned populations of different functional arrays within the same sample chamber. For this, we inhibited the formation of a single extended uniform array throughout the chamber by limiting local molecular diffusion on mixing PDDA (100–200 kDa) and ATP. This was achieved by reversing the standard location of the PDDA and ATP solutions such that ATP was contained within the sample chamber and a small volume of PDDA along with an enzyme then gently injected at a localized position and the mixture left undisturbed in the acoustic field. Consequently, the ATP: PDDA monomer molar ratio was 100: 1 rather than 1: 1, and coacervation occurred immediately around the point of injection to produce primary droplets that coalesced in the acoustic field to generate a highly localized array of enzyme-containing single droplets (Supplementary Fig. 15). By repeating this procedure but using three different enzymes with relatively high partition constants (*K*) (GOx, *K*=60; amyloglucosidase (AGx, *K*=355); or horseradish peroxidase (HRP), *K*=62)) in the injected PDDA solutions, we were able to prepare distinct spatial domains of functional droplet arrays within the same device (Fig. 5a).

Given the above observations, we reasoned that it should be possible to develop acoustic trapping protocols for the preparation of water-based droplet arrays capable of sensing specific chemicals in the environment. As proof-of-principle we acoustically patterned an array of HRP-containing PDDA/ATP coacervate micro-droplets at a spacing of *ca.* 110 μm, and exposed the pattern to a gradient of H_2_O_2_ and *o*-phenylenediamine (*o*-PD) by diffusing the substrate mixtures specifically from one side of the device. Conversion of non-fluorescent *o*-PD to fluorescent cationic 2,3-diaminophenazine (2,3-DAP) by the coacervate droplet-sequestered enzyme was recorded by fluorescence microscopy^42^,^43^. Within a few minutes of injecting the small molecule substrates the fluorescence images showed a clear response to the environmental stimulus in the form of a chemical wavefront that propagated through the coacervate micro-droplet array (Fig. 5b). Measurements of the fluorescence associated with HRP-mediated 2,3-DAP production in individual coacervate droplets positioned along a single row lying parallel to the direction of diffusion showed a lag time of *ca*. 12 s between adjacent droplets (Fig. 5c). Line profiles recorded along the direction of diffusion at different time intervals showed that the output signals of each droplet were strongly dependent on their spatial positions (Fig. 5d). Moreover, although the droplets were activated at different times depending on their proximity to the diffusion front, the rate of change in mean fluorescence intensity was similar for each droplet (Fig. 5c), suggesting that the enzymatic response was effectively unchanged across the 2D array. In contrast, only marginal differences in activation were observed between adjacent droplets positioned in single rows lying perpendicular to the diffusion front (Fig. 5e).

## Discussion

Our results indicate that structured acoustic radiation forces are a powerful, versatile and inexpensive tool to manipulate the spatial assembly of uniform-sized coacervate micro-droplets in aqueous media to produce functional water-based molecularly crowded liquid droplet periodic arrays comprising selective chemicals, biomolecules and catalysts. Patterning functional water-based droplets in aqueous media is challenging because of the minimal acoustic contrast, structural instability associated with low interfacial tension, and difficulty of sequestering solutes into the droplets due to the small values of the equilibrium partitioning constants. These challenges have been circumvented by combining an acoustic standing wave force field with *in situ* complex coacervation to generate defect-free arrays of single coacervate droplets or droplet aggregates arranged in 2D lattices with controllable spacing, variable surface-attachment properties and reversible dynamical behaviour. The final size of the droplets can be controlled by changes in the chemical concentrations or by quenching coalescence of the primary droplets to produce 2D arrays with fixed spacing and geometry but variable droplet size. While our studies have focused on the generation of square grids, it should be straightforward to extend the methodology to more complex droplet patterns through appropriate geometrical reconfiguration of the piezoelectric transducers and sample chamber^44^.

A key advantage of the described methodology is associated with the diverse physical and chemical properties of coacervates, which can be readily tailored by changes in composition, binding constants, dehydration and surface charge^17^,^18^,^19^,^20^,^21^,^22^,^23^. As a consequence, coacervate micro-droplets exhibiting variable viscosities, different levels of molecular crowding and surface adherence, and diverse sequestration of small molecules and macromolecules can be easily prepared using a wide range of components and conditions. Acoustic patterning of such droplets could, therefore, provide steps towards new types of aqueous-based arrays for use in the storage and spatially ordered release of drugs such as antimicrobials from 2D platforms, as ordered substrates for the patterning of micro-reactors based on DNA/enzyme sensing, and as organized platforms for biomolecule purification and protein crystallization. In each case, the high sequestration potential and relatively low dielectric constant of the molecularly crowded coacervate droplets specifically enable the selective uptake, storage and potential communication of a wide range of functional components capable of operating dynamically in a continuous aqueous media.

Finally, we describe a rudimentary demonstration of using chemically encoded droplet arrays for the enzyme-mediated sensing of substrate molecules flowing through the sample chamber, suggesting that it should be possible to develop devices capable of sustaining chemical signals between the droplets as well as enabling spatial and temporal responses to changing conditions in the external environment. While this is a challenging prospect, the ability to spatially position multiple populations of functionally correlated droplets under water within the same device, such as three members of an enzyme cascade reaction as shown in Fig. 5a, control their surface attachment, and acoustically regulate their inter-droplet edge distance to control the diffusion length of signalling molecules could together provide the basis for increased operational complexity. In this regard, we note that coacervate micro-droplets have been recently exploited as models of membrane-free protocells^24^,^25^,^26^,^27^,^28^,^29^, suggesting that the combination of acoustic patterning and *in situ* coacervation could provide a novel route towards the design and construction of protocell communities with controllable spatial organization, programmable signalling pathways and chemical circuitry, and higher order collective behaviour.

## Methods

### Acoustic trapping

A custom-built acoustic trapping device based on a square arrangement of four piezoelectric transducers with a thickness of 1 mm (Noliac, NCE 51, L15 × W2 mm) or 0.4 mm (Noliac, NCE 51, L15 × W5 mm) was used. The opposing transducer pairs were wired in series, driven by two signal generators (Agilent 33220a-001), and connected to an oscilloscope (Agilent DSOX2014A). The orthogonal transducer pairs were run at slightly different frequencies during the trapping process. Transducer pairs with a thickness of 1 or 0.4 mm were operated at the third harmonic frequency (6.76/6.78 MHz) or at their fundamental frequency (4.99/5.00 MHz), respectively. The corresponding wavelengths were 219/218 μm (6.76/6.78 MHz) and 297/296 μm (4.99/5.00 MHz).

### Acoustic-mediated assembly and geometric patterning

Neutrally charged PDDA/ATP coacervate micro-droplets were prepared *in situ* within a custom-built acoustic trapping device based on a square arrangement of four piezoelectric transducers. Typically, an aqueous solution of PDDA (1 ml, 5 mM monomer, 100–200 kDa) was placed into the device chamber and aqueous ATP (100 μl, 50 mM) then added in the presence of two orthogonal acoustic standing waves generated from opposing transducer pairs operating at 6.76/6.78 MHz (10 V). Experiments were also undertaken under the same field conditions but at different polymer and nucleotide concentrations (PDDA; 1 ml, 2.5 and 7.5 mM monomer, 100–200 kDa; ATP; 100 μl , 25 and 75 mM). Solution volumes were doubled (PDDA, 2 ml, 5 mM monomer; ATP, 200 μl, 50 mM) for trapping experiments undertaken with transducer pairs operating at lower frequencies of 4.99/5.00 MHz. In all cases, the PDDA monomer: ATP molar ratio was approximately 1: 1, mixtures were stirred to ensure homogeneous formation of the coacervate droplets in the square chamber, and the pH was adjusted to 7. The composition of the trapped coacervate micro-droplets was elucidated using fluorescence microscopy by doping the polymer/nucleotide mixtures with a fluorescent derivative of ATP (TNP-ATP) or a rhodamine-tagged cationic polymer (RITC-PAH). Samples of doped PDDA/ATP droplets were prepared with a TNP-ATP: ATP or PAH: PDDA monomer molar ratios of 1:1000, or 1:9, respectively.

Similar procedures were used to self-assemble, trap and acoustically pattern coacervate PDDA-containing coacervate micro-droplets prepared from a range of components including proteins (BSA), polysaccharides (CM-D), polynucleotides (dsDNA) and anionic polymers (PAA), as well as coacervates formed by complexation of PEI and CM-D. In each case, solutions of BSA (1 ml, 5 mg ml^−1^, pH 7), CM-D (1 ml, 45 mM monomer, pH 8), 4/1 (mol/mol) CM-D/FITC-CM-D mixtures (1 ml, 45 mM total monomer, pH 8), dsDNA (1 ml, 5 mg ml^−1^, Tris buffer (10 mM, pH 8)) or PAA (100 μl, 200 mM monomer, pH 8) were placed in the chamber of the acoustic device operating with two orthogonal acoustic standing waves (6.76/6.78 MHz, 10 V), and the following amounts of PDDA with a molecular weight of 8.5 kDa added: BSA (62 μl, 50 mM monomer, pH 7), CM-D (75 μl, 100 mM monomer, pH 8), 4/1 (mol/mol) CM-D/FITC-CM-D mixtures (75 μl, 100 mM monomer, pH 8), dsDNA (100 mM, 150 μl, 8.5 kDa, monomer, Tris buffer (10 mM, pH=8)) or PAA (1 ml, 20 mM monomer, pH 8). Typically, a PDDA: BSA weight ratio of 1: 10, and PDDA: CM-D, PDDA: DNA base, and PDDA: PAA monomer molar ratios of approximately 1: 6, 1: 1 and 1: 1, respectively, were used. PEI/CM-D coacervate droplets were prepared by injecting an aqueous solution of PEI (50 μl, 300 mM monomer) into a CM-D solution (1 ml, 15 mM monomer) housed in the chamber of the acoustic device operating with two orthogonal acoustic standing waves (6.76/6.78 MHz, 10 V) to give a final monomer molar ratio of 1: 1 and pH 8. In each case, the mixtures were stirred to ensure a homogeneous formation of coacervate droplets in the square chamber.

### Uptake studies in PDDA/ATP droplet arrays

Acoustically patterned arrays of PDDA/ATP droplets were prepared as above (6.76/6.78 MHz, 10 V) in the presence of methylene blue (10 μl, 1 mM), nile red (10 μl, 1 mM), sulforhodamine B (10 μl, 1 mM) or calcein (1 μl, 1 mM), and fluorescence images recorded after 15 min to determine the level of molecular uptake. Sequestration of polymer or inorganic particles into the acoustically trapped droplets was undertaken by injecting 100 μl of premixed polystyrene nanoparticles (100 nm, 5 × 10^−2^ wt.%) or silica microparticles (2.5 μm, 2.5 × 10^−2^ wt.%) and aqueous ATP (100 μl, 50 mM) into a PDDA solution (1 ml, 5 mM monomer; PDDA monomer: ATP=1, pH =7) contained with the device and subjected to two orthogonal acoustic standing waves (6.76/6.78 MHz, 10 V). The mixtures were stirred to ensure homogeneous formation of the coacervate droplets in the square chamber. Three-dimensional confocal microscopy construction of the silica microparticle-containing PDDA/ATP droplets was undertaken by adding sulforhodamine B (10 μl, 1 mM) to the coacervate micro-droplet array after 45 min of acoustic processing. *In situ* sequestration of FITC-GOx into PDDA/ATP acoustically trapped micro-droplet arrays was achieved by injecting aqueous ATP (100 μl, 50 mM) into 1 ml of PDDA (8.5 kDa, 5 mM monomer) containing FITC-GOx (1 μg ml^−1^) in the presence of two orthogonal acoustic standing waves (6.76/6.78 MHz, 10 V; PDDA monomer: ATP=1, pH=7). Partition coefficients (*K*) for dye molecules and enzymes were determined by measuring the concentrations (*C*) in the supernatant (super) and coacervate (coac) phase, and given as *K*=*C*_coac_*/C*_super._ Concentrations were determined from characteristic absorption/emission spectra according to previously reported methods^25^.

### Spatial organization of acoustically trapped PDDA/ATP droplet arrays

A 1 μl volume of a pre-mixed solution of PDDA (25 mM monomer, 100–200 kDa) and FITC-GOx (0.2 mg ml^−1^) were gently added to an ATP solution (1 ml, 2.5 mM) contained within the chamber of an acoustic trapping device in the presence of two orthogonal acoustic standing waves (6.76/6.78 MHz, 10 V). After injection, the device was left undisturbed so that enzyme-containing coacervate micro-droplets were formed specifically at the nodal regions within a localized area close to the point of injection of the PDDA/FITC-GOx mixture. A mixture (1 μl) of PDDA (25 mM monomer) and RITC-HRP (0.2 mg ml^−1^) was then injected under the same acoustic standing wave field at a different location in the ATP-filled chamber so that two spatially separated arrays of PDDA/ATP droplets containing either FITC-GOx or RITC-HRP were obtained. Repeating this procedure with a mixture of PDDA (1 μl, 25 mM monomer) and Dylight 405-AGx (0.2 mg ml^−1^) produced a third population of PDDA/ATP droplets within the device chamber.

### Enzyme reactions in acoustically trapped droplets

A mixture (1 μl) of PDDA (25 mM monomer, 100–200 kDa) and RITC-HRP (0.2 mg ml^−1^) was added to 1 ml of ATP (2.5 mM) in the presence of two orthogonal acoustic standing waves (6.76/6.78 MHz, 10 V). After 45 min, the acoustic field was switched off and then the supernatant was carefully removed and exchanged with Milli-Q water three times without disturbing the array of coacervate micro-droplets. 20 μl of a mixture of H_2_O_2_ (50 mM) and *o*-phenylenediamine (*o*-PD, 25 mM) was then added at the bottom of the device, and left undisturbed. Fluorescence microscopy (*λ*_ex_=355–425 nm, *λ*_em_=455 nm) was used to detect the HRP-mediated conversion of non-fluorescence *o*-PD to fluorescence cationic 2,3-DAP.

### Simulation methods

Each opposed transducer pair is excited with a sinusoidal voltage and after an initial transient period an acoustic standing wave is established in the chamber. If reflections are ignored, the standing wave can be thought of as the sum of two counter-propagating plane waves according to a previous approach^45^. The total acoustic pressure in the devices is then given by the sum of the two standing waves created by the orthogonal pairs:


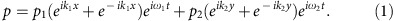


where 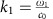 and *ω*_1_ is the angular frequency in rad/s and *c*_0_ is the speed of sound in the host fluid. The numerical subscript denotes the orthogonal pair under consideration, and is required as the transducer pairs were often operated at different frequencies. The Cartesian axes are defined by *x* and *y*. Transducer pair-1 creates a standing wave in *x* whereas transducer pair-2 creates a standing wave in *y*. Gor'kov described the forces as resulting from a potential field, *U*. Using this approach the acoustic radiation force, 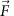, can be found from


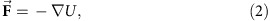



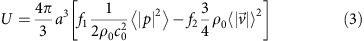



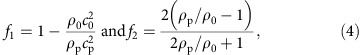


where 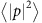 and 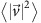 are the mean squared pressure and particle velocity respectively at the object, *a* is the radius of the spherical object, *ρ* is the density and the subscripts denote the particle, ‘*p*', or host, ‘o' properties. Note also, for a harmonic sound field, 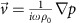.

In devices where the transducers were operated at the same frequency, equation (1) was applied directly to the resultant pressure field. In cases where each of the transducer pairs were operated at different frequencies, equation (1) was applied to the field from each pair separately and the results summed. This latter calculation assumes that any interference between these two fields averages to zero, which is the case for time periods significantly greater than the modulation time, 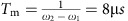.

### Data availability

All underlying data are included in full within this paper and in the supplementary information.

## Additional information

**How to cite this article:** Tian, L. *et al*. Spontaneous assembly of chemically encoded two-dimensional coacervate droplet arrays by acoustic wave patterning. *Nat. Commun.* **7,** 13068 doi: 10.1038/ncomms13068 (2016).

## Supplementary Material

Supplementary Information Supplementary Figures 1-15 and Supplementary Methods

Supplementary Movie 1 Optical microscopy video showing growth of acoustically trapped PDDA/ATP coacervate micro-droplets in an aqueous medium via coalescence of primary droplets at the acoustic nodes. The droplets were prepared by injecting an aqueous solution of ATP (100 μL, 25 mM) into a PDDA solution (1 mL, 2.5 mM monomer, 100-200 kDa) contained within the sample chamber of the acoustic trapping device in the presence of two orthogonal transducer pairs operating at 6.76/6.78 MHz (10 V). Movie is shown at x80 of real-time speed at 16 frames per second. Total duration of recording was 45 minutes in real time.

Supplementary Movie 2 Optical microscopy video showing an array of PDDA/CM-D coacervate micro-droplets displaying spatially confined localized spinning in the aqueous medium contained within the sample chamber of an acoustic trapping device in the presence of two orthogonal transducer pairs operating at 6.76/6.78 MHz (10 V). The array of PDDA/CM-D coacervate micro-droplets was prepared by adding PDDA (75 μL, 100 mM monomer, 8.5 kDa) to a CMD (1 mL, 45 mM monomer) aqueous solution contained within the sample chamber of an acoustic trapping device operated under the acoustic standing wave field. Movie is shown at x100 of real-time speed at 20 frames per second. Total duration of recording was 15 minutes in real time.

Supplementary Movie 3 Optical microscopy video showing reversible modulation in an aqueous medium of the spatial configuration of localized aggregates of sub-micrometre-sized PDDA/FITC-CMD/CM-D droplets by changing the frequencies of the two orthogonal acoustic standing waves from 6.76/6.78 MHz to 6.76/6.76 MHz. The droplets were initially prepared by adding PDDA (100 μL, 50 mM monomer, 8.5 kDa) to 1 mL of a CM-D (36 mM)/FITC-CM-D (9 mM) mixture contained within the sample chamber of an acoustic trapping device using two transducer pairs operating at 6.76/6.78 MHz (10 V). Movie is shown at x1 of real-time speed at 27 frames per second. Total duration of recording was 8.4 seconds in real time.

Supplementary Movie 4 Fluorescence microscopy video showing reversible transformation of localized spherical aggregates of sub-micrometre-sized PDDA/FITC-CM-D/CM-D droplets in an aqueous medium into vertically or horizontally oriented elliptical shapes and continuous fringes by switching on/off the corresponding pairs of piezoelectric transducers. The droplets were prepared by adding PDDA (100 μL, 50 mM monomer, 8.5 kDa) to 1 mL of a CM-D (36 mM)/FITC-CM-D (9 mM) mixture contained within the sample chamber of an acoustic trapping device using two transducer pairs operating at 6.76/6.78 MHz (10 V). Movie is shown at x10 of real-time speed at 7.7 frames per second. Total duration of recording was 95 seconds in real time.
