# Supplementary material for: Spontaneous assembly of chemically encoded two-dimensional coacervate droplet arrays by acoustic wave patterning
Source: Nat Commun. 2016 Oct 6;7:13068. doi: 10.1038/ncomms13068 (PMC5059748; doi:10.1038/ncomms13068)
Supplement: Supplementary Information — Supplementary Figures 1-15 and Supplementary Methods [file ncomms13068-s1.pdf]

Supplementary Information

Supplementary Figures

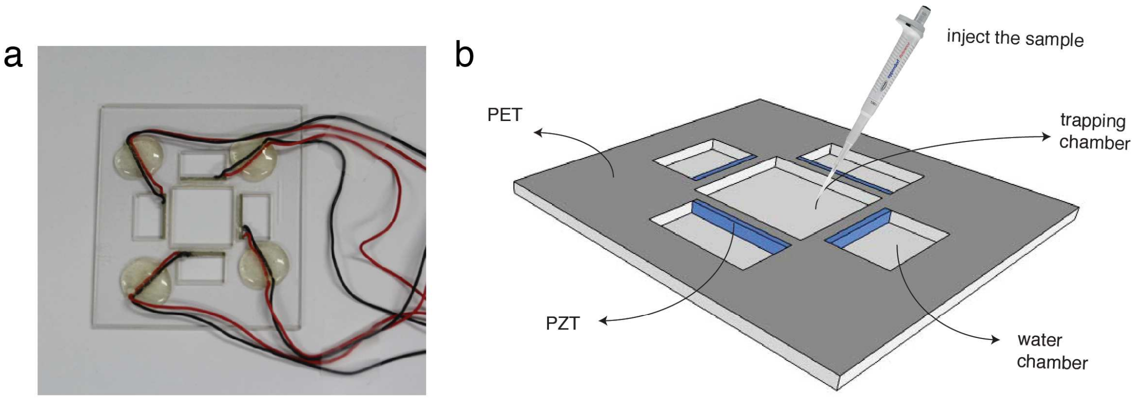

Supplementary Figure 1. (*a*-*b*) Optical image (top view) and schematic representation of the custom-made acoustic trapping device. The device was constructed from poly(ethylene terephthalate) (PET) and four piezoelectric transducers (blue cuboids as shown in (*b*)) oriented as two orthogonal pairs. Two different kinds of piezoelectric transducers were used with a thickness of 1 mm (Noliac, NCE 51, L15 x W2 mm) or a thickness of 0.4 mm (Noliac, NCE 51, L15 x W5 mm). Depending on the width of transducers, 2 mm and 5 mm thickness of PET sheets were applied accordingly. Each transducer was attached with adhesive to the back of the central square chamber (20 x 20 mm) with cyanoacrylate, and the four rectangular chambers surrounding the central sample chamber were used to store water to provide cooling. A glass coverslip was attached with adhesive to the bottom of the device. The trapping device had an open chamber, and the fluid was injected into the acoustic trapping chamber using an eppendorf pipette. After injection, coacervate micro-droplets were spontaneously assembled and patterned into 2D arrays within the chamber due to the periodic acoustic standing wave pressure field. The device was re-used either by replacing the glass coverslip, or by washing the chamber with NaCl solution (0.5 M). Various chemicals could be injected into the sample chamber without disturbing the preformed droplet arrays using an eppendorf pipette.

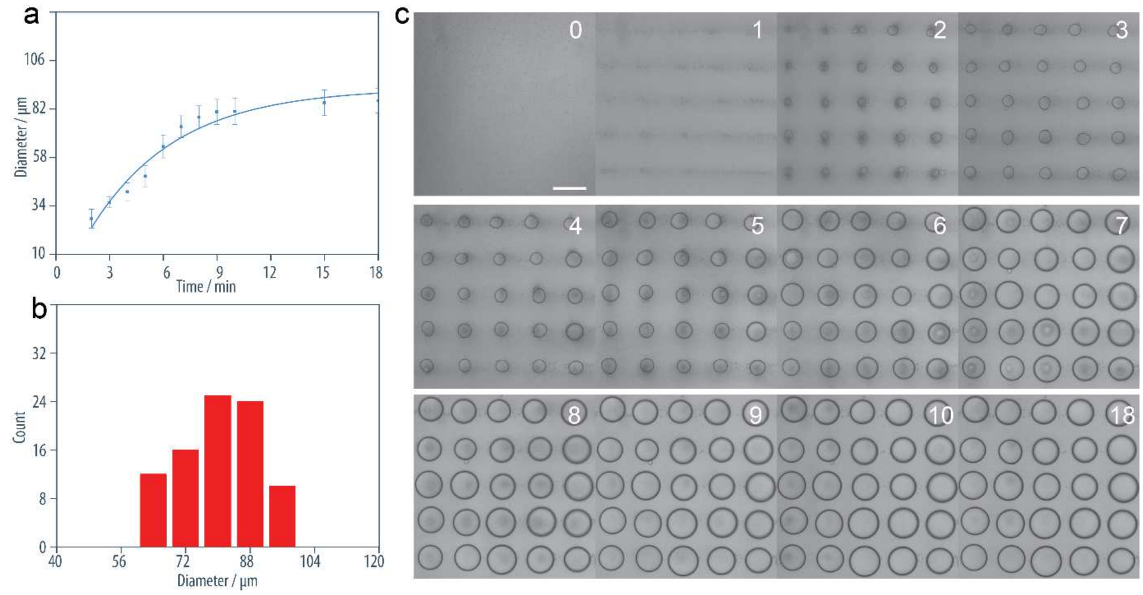

Supplementary Figure 2. (*a*) Plot showing time-dependent changes in mean diameter of PDDA/ATP micro-droplets located at the nodes of an acoustic standing wave pressure field after mixing PDDA and ATP. The PDDA/ATP droplets were prepared by adding ATP (100 µL, 75 mM) to a PDDA (1 mL, 7.5 mM, monomer, 100-200 kDa) solution contained within the sample chamber of an acoustic trapping device operated under two orthogonal acoustic standing waves (6.76/6.78 MHz, 10V). (*b*) Size distribution of PDDA/ATP droplets produced 18 min after mixing of PDDA and ATP under the above conditions. (*c*) Time-dependent optical microscopy images showing growth of PDDA/ATP coacervate droplets at the nodes of an acoustic standing wave pressure field; the images were recorded at minute intervals between 1-10 min, and 18 min after mixing PDDA and ATP under the above conditions; scale bar = 100 µm.

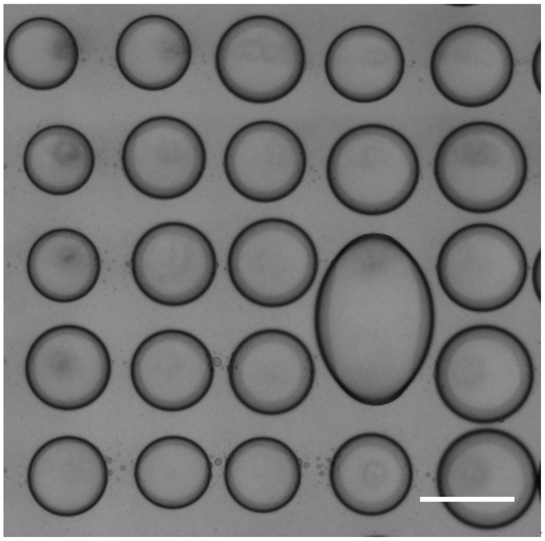

Supplementary Figure 3. Optical microscopy image showing fusion of two adjacent PDDA/ATP coacervate droplets in a patterned array produced under an acoustic standing wave field (6.76/6.78 MHz, 10 V). Merging of individual droplets occurred 10 min after mixing PDDA (1 mL, 7.5 mM, monomer, 100-200 kDa) and ATP (100 µL, 75 mM) in the acoustic device; scale bar = 100 µm.

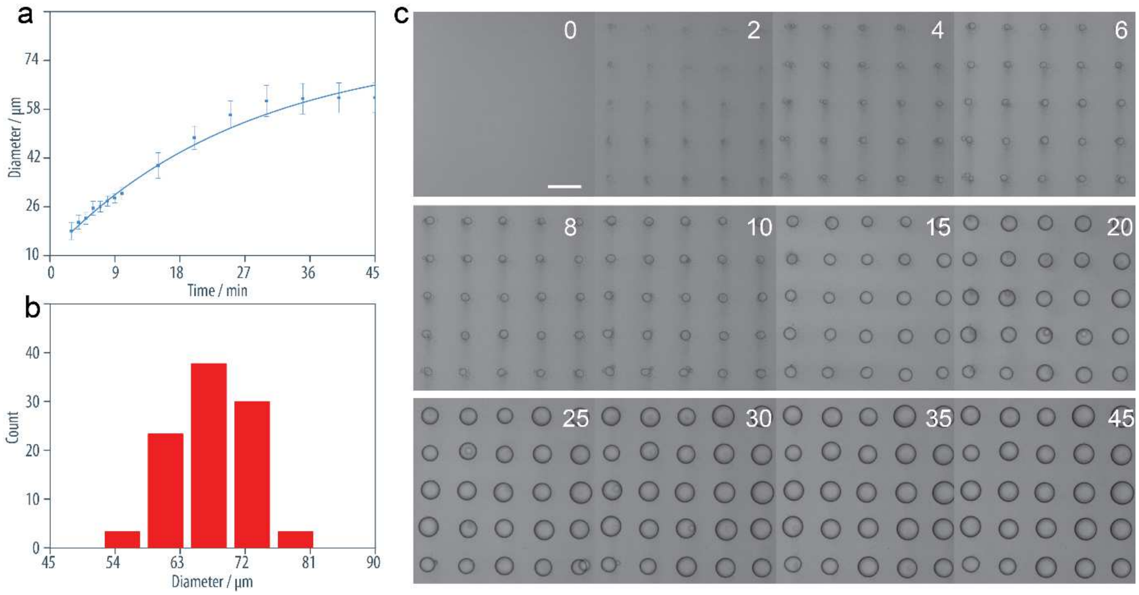

Supplementary Figure 4. (*a*) Plot showing time-dependent changes in mean diameter of PDDA/ATP micro-droplets located at the nodes of an acoustic standing wave pressure field after mixing PDDA and ATP. The PDDA/ATP droplets were prepared by adding ATP (100 µL, 25 mM) to a PDDA (1 mL, 2.5 mM monomer, 100-200 kDa) solution contained within the sample chamber of an acoustic trapping device operated under two orthogonal acoustic standing waves (6.76/6.78 MHz, 10 V). (*b*) Size distribution of PDDA/ATP droplets produced 45 min after mixing of PDDA and ATP under the above conditions. The mean size after 15 min was *ca*. 40 µm (final [ATP] = 2.5 mM) compared with 64 µm for droplets prepared at 5 mM ATP and left to coalesce in the acoustic field for 25 min. (*c*) Time-dependent optical microscopy images showing growth of PDDA/ATP coacervate droplets at the nodes of an acoustic standing wave pressure field; the images were recorded at 0, 2, 4, 6, 8, 10, 15, 20, 25, 30, 35 and 45 min after mixing PDDA and ATP under the above conditions; scale bar = 100 µm.

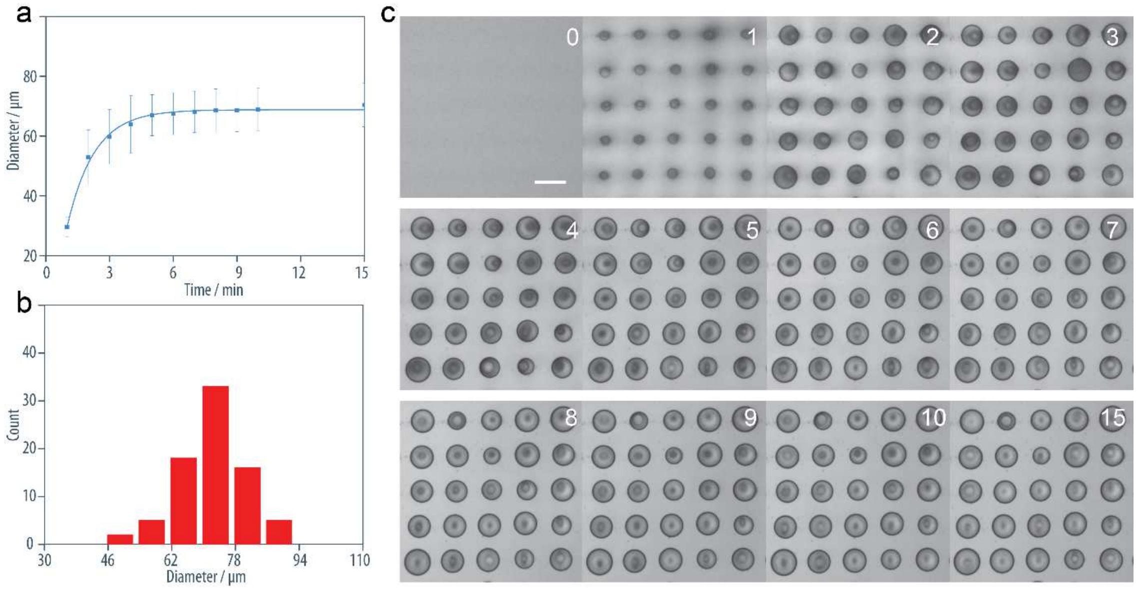

Supplementary Figure 5. (*a*) Plot showing time-dependent changes in mean diameter of PDDA/PAA micro-droplets located at the nodes of an acoustic standing wave pressure field after mixing PDDA and PAA. The PDDA/PAA droplets were prepared by adding PDDA (1 mL, 20 mM monomer, 8.5 kDa) to a PAA (100 µL, 200 mM) solution contained within the sample chamber of an acoustic trapping device operated under two orthogonal acoustic standing waves (6.76/6.78 MHz, 10 V). (*b*) Size distribution of PDDA/PAA droplets produced 15 min after mixing of PDDA and PAA under the above conditions. (*c*) Time-dependent optical microscopy images showing growth of PDDA/PAA coacervate droplets at the nodes of an acoustic standing wave pressure field; the images were recorded at minute intervals between 0-10 min and 15 min after mixing PDDA and PAA under the above conditions; scale bar = 100 µm.

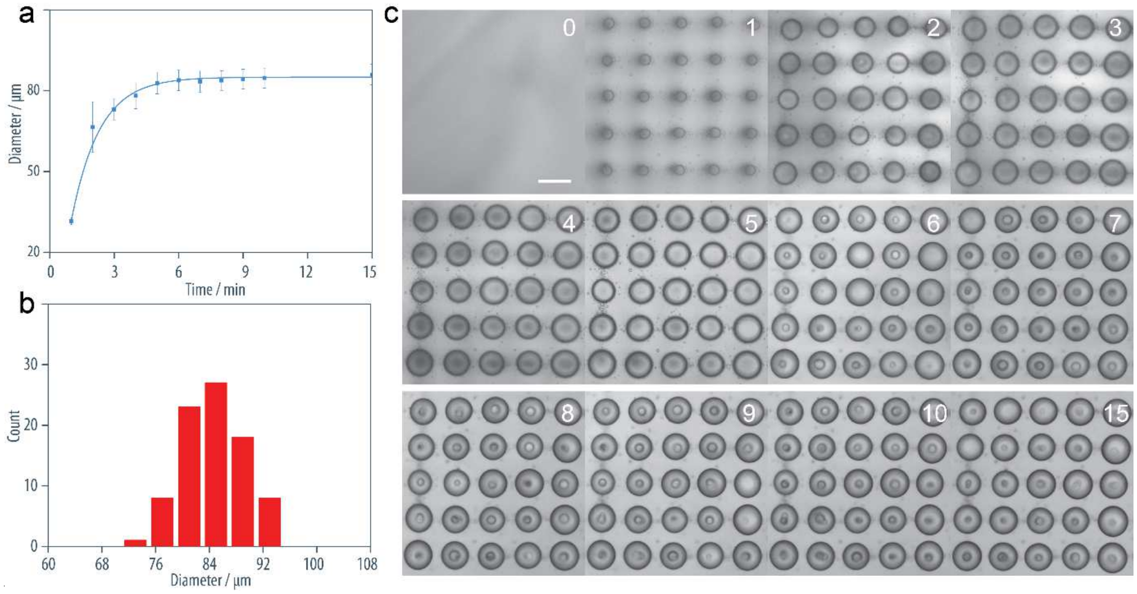

Supplementary Figure 6. (*a*) Plot showing time-dependent changes in mean diameter of PDDA/DNA micro-droplets located at the nodes of an acoustic standing wave pressure field after mixing PDDA and dsDNA. The PDDA/DNA droplets were prepared by adding PDDA (150 µL, 100 mM monomer, 8.5 kDa, Tris buffer (10 mM, pH 8)) to a DNA (1 mL, 5 mg mL^{-1}, Tris buffer (10 mM, pH 8)) solution contained within the sample chamber of an acoustic trapping device operated under two orthogonal acoustic standing waves (6.76/6.78 MHz, 10 V). The dsDNA was dissolved in the buffer by heating at 80°C h for 6 h and then slowly cooled to room temperature. (*b*) Size distribution of PDDA/DNA droplets produced 15 min after mixing of PDDA and DNA under the above conditions. (*c*) Time-dependent optical microscopy images showing growth of PDDA/DNA coacervate droplets at the nodes of an acoustic standing wave pressure field; the images were recorded at minute intervals between 0-10 min and at 15 min after mixing PDDA and DNA under the above conditions; scale bar = 100 µm.

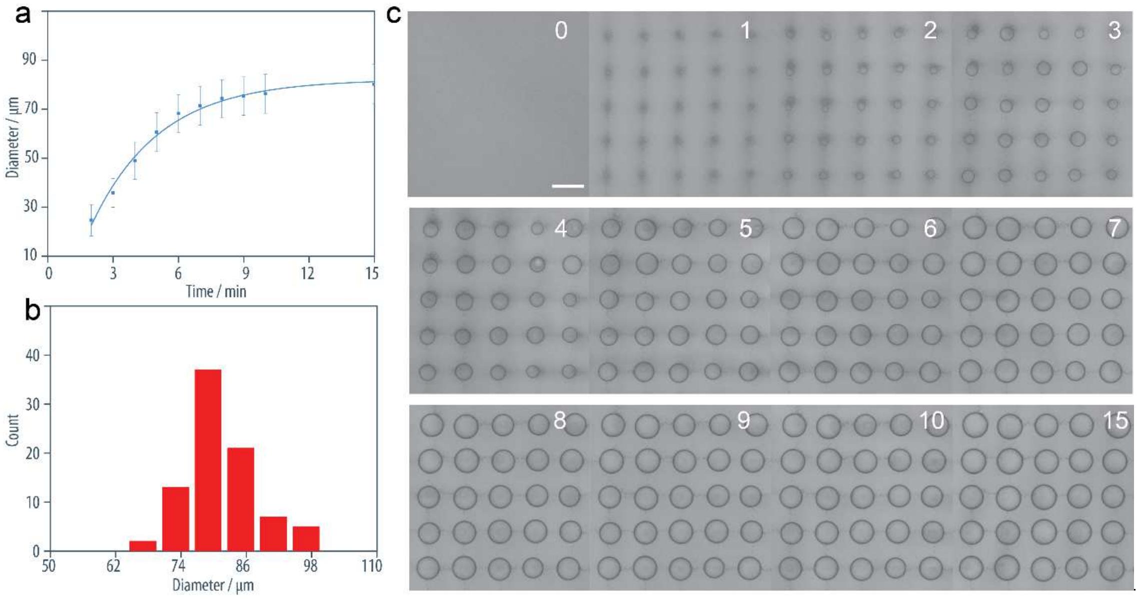

Supplementary Figure 7. (*a*) Plot showing time-dependent changes in mean diameter of PDDA/CM-D micro-droplets located at the nodes of an acoustic standing wave pressure field after mixing PDDA and CM-D. The PDDA/CM-D droplets were prepared by adding PDDA (75 µL, 100 mM monomer, 8.5 kDa) to a CM-D (1 mL, 45 mM monomer) solution contained within the sample chamber of an acoustic trapping device operated under two orthogonal acoustic standing waves (6.76/6.78 MHz, 10 V). (*b*) Size distribution of PDDA/CM-D droplets produced 15 min after mixing of PDDA and CM-D under the above conditions. (*c*) Time-dependent optical microscopy images showing growth of PDDA/CM-D coacervate droplets at the nodes of an acoustic standing wave pressure field; the images were recorded at minute intervals between 0-10 min and at 15 min after mixing PDDA and CM-D under the above conditions; scale bar = 100 µm.

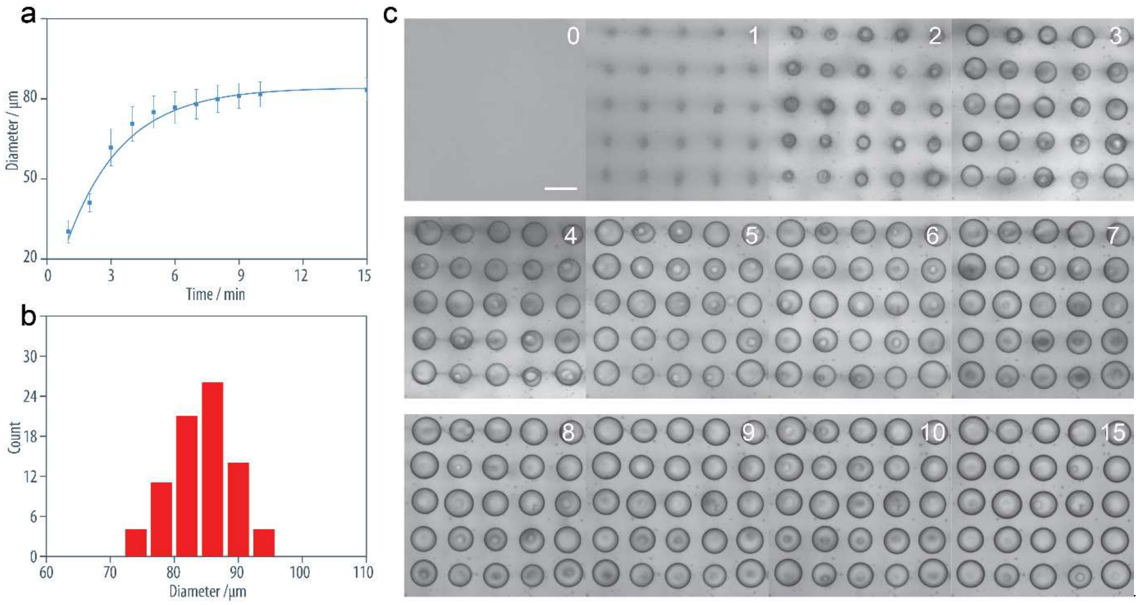

Supplementary Figure 8. (*a*) Plot showing time-dependent changes in mean diameter of PDDA/BSA micro-droplets located at the nodes of an acoustic standing wave pressure field after mixing PDDA and BSA. The PDDA/BSA droplets were prepared by adding PDDA (62 µL, 50 mM monomer, 8.5 kDa) to a BSA (1 mL, 5 mg mL^{-1}) solution contained within the sample chamber of an acoustic trapping device operated under two orthogonal acoustic standing waves (6.76/6.78 MHz, 10 V). (*b*) Size distribution of PDDA/CM-D droplets produced 15 min after mixing of PDDA and BSA under the above conditions. (*c*) Time-dependent optical microscopy images showing growth of PDDA/BSA coacervate droplets at the nodes of an acoustic standing wave pressure field; the images were recorded at minute intervals between 0-10 min and at 15 min after mixing PDDA and BSA under the above conditions; scale bar = 100 µm.

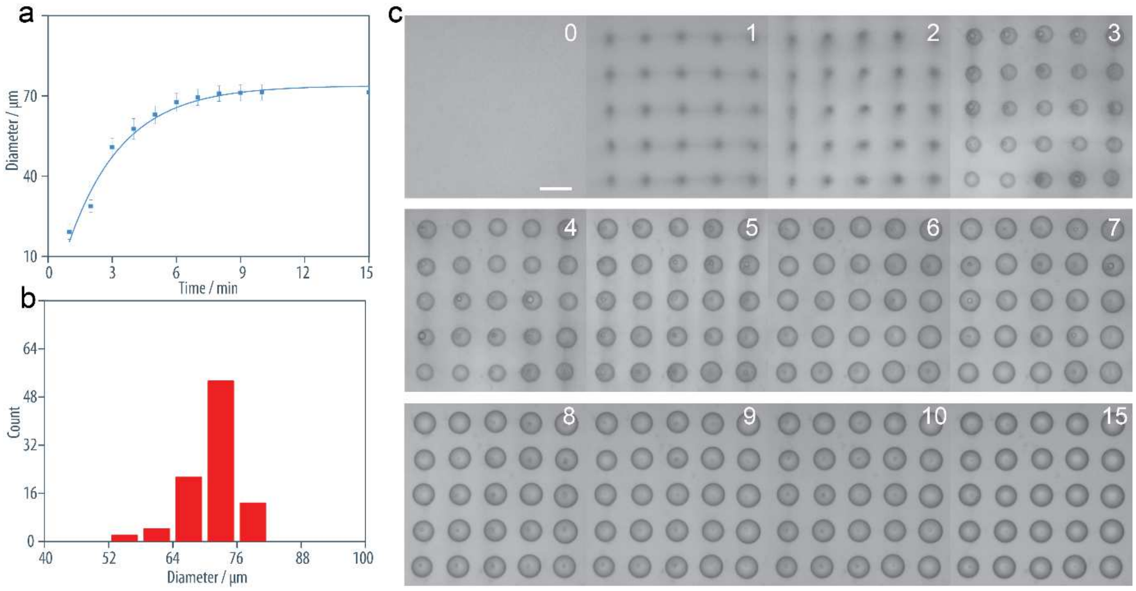

Supplementary Figure 9. (*a*) Plot showing time-dependent changes in mean diameter of PEI/CM-D micro-droplets located at the nodes of an acoustic standing wave pressure field after mixing PEI and CM-D. The PEI/CM-D droplets were prepared by adding PEI (50 µL, 300 mM monomer) to a CM-D (1 mL, 15 mM monomer) solution contained within the sample chamber of an acoustic trapping device operated under two orthogonal acoustic standing waves (6.76/6.78 MHz, 10 V). (*b*) Size distribution of PEI/CM-D droplets produced 15 min after mixing of PEI and CM-D under the above conditions. (*c*) Time-dependent optical microscopy images showing growth of PEI/CM-D coacervate droplets at the nodes of an acoustic standing wave pressure field; the images were recorded at minute intervals between 0-10 min and at 15 min after mixing PEI and CM-D under the above conditions; scale bar = 100 µm.

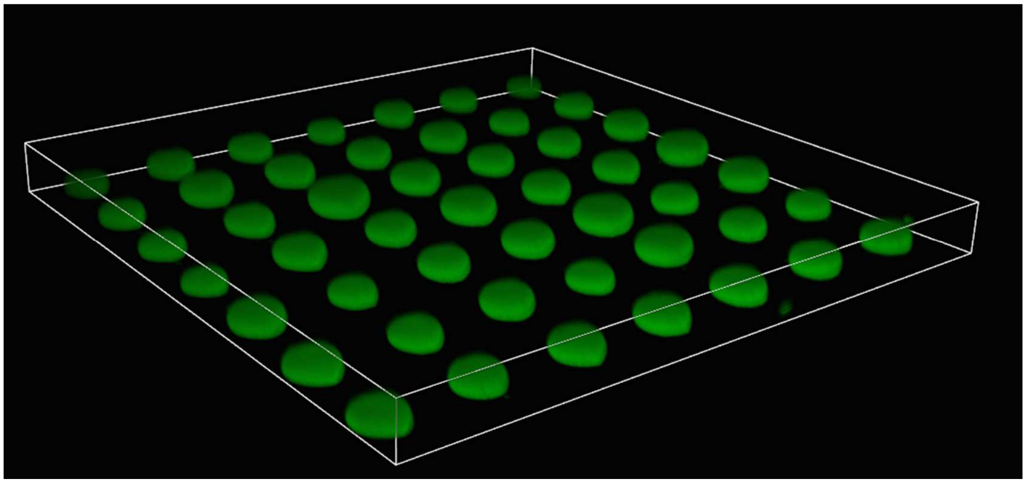

Supplementary Figure 10. 3D confocal fluorescence microscopy image of acoustically trapped TNP-ATP-doped PDDA/ATP droplets showing strong attachment to the underlying PEGylated glass substrate of the channel chamber and associated hemispherical morphology. The PDDA/ATP/TNP-ATP droplets were prepared by adding a mixture of TNP-ATP (0.1 mol%) and ATP (100 µL, 50 mM) to a PDDA solution (1 mL, 5.0 mM monomer, 100-200 kDa) contained within the sample chamber of an acoustic trapping device operated under two orthogonal acoustic standing waves (6.76/6.78 MHz, 10 V).

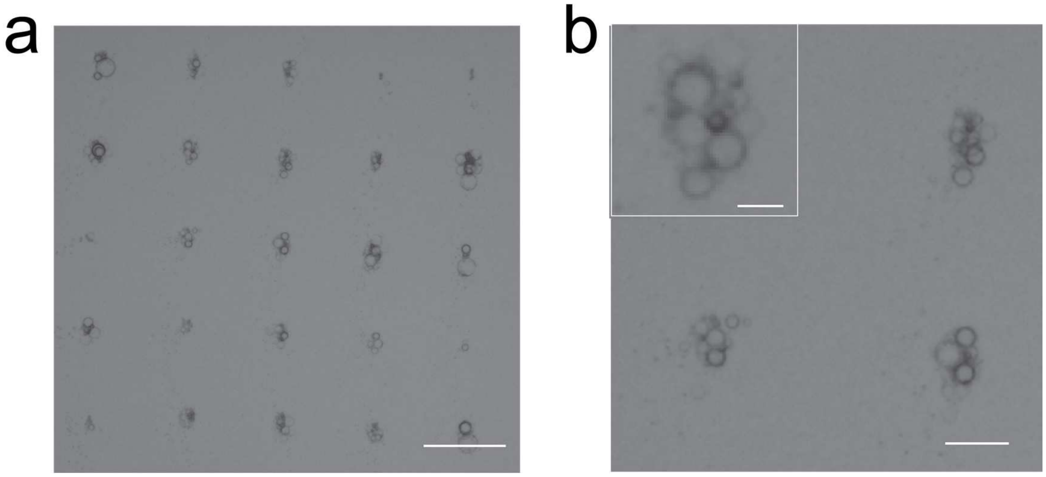

Supplementary Figure 11. Optical microscopy images of acoustically patterned PDDA/BSA droplet aggregates produced using transducer pairs operated at 6.76 and 6.78 MHz (10 V) and recorded after 30 min. The droplets were prepared by addition of PDDA (62 µL, 50 mM monomer, 100-200 kDa) to BSA (1 mL, 5 mg mL^{-1}) contained in the sample holder. (*a*) Low magnification image showing 2D array of droplet aggregates; scale bar = 100 µm. (*b*) Higher magnification image showing three sets of trapped droplet aggregates; inset showing single aggregate with non-coalescing droplets; scale bars = 50 µm and 10 µm (inset). No coalescence of the primary droplets was observed after 30 min.

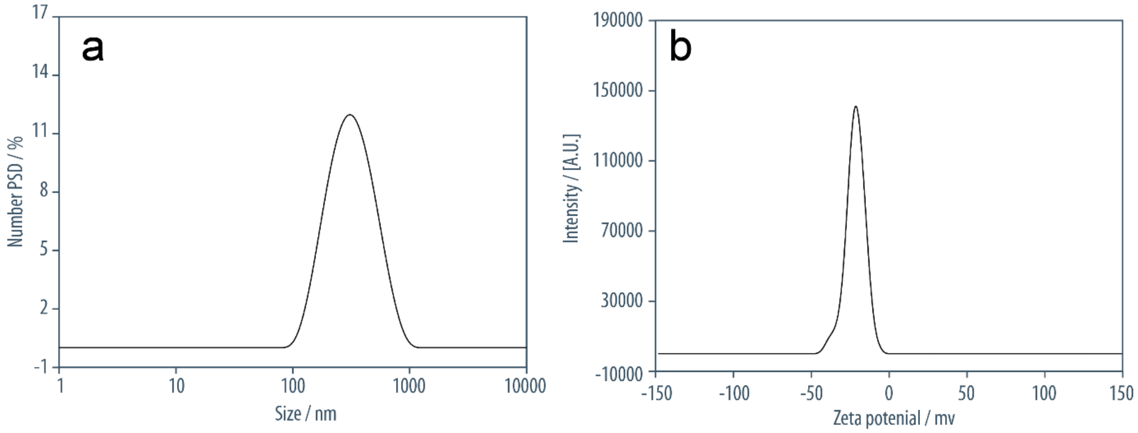

Supplementary Figure 12. (*a*) Dynamic light scattering size distribution and (*b*) zeta potential profile for non-coalescing sub-micrometre PDDA/FITC-CM-D/CM-D droplets. Mean diameter = 310 nm; surface potential = -21 mV.

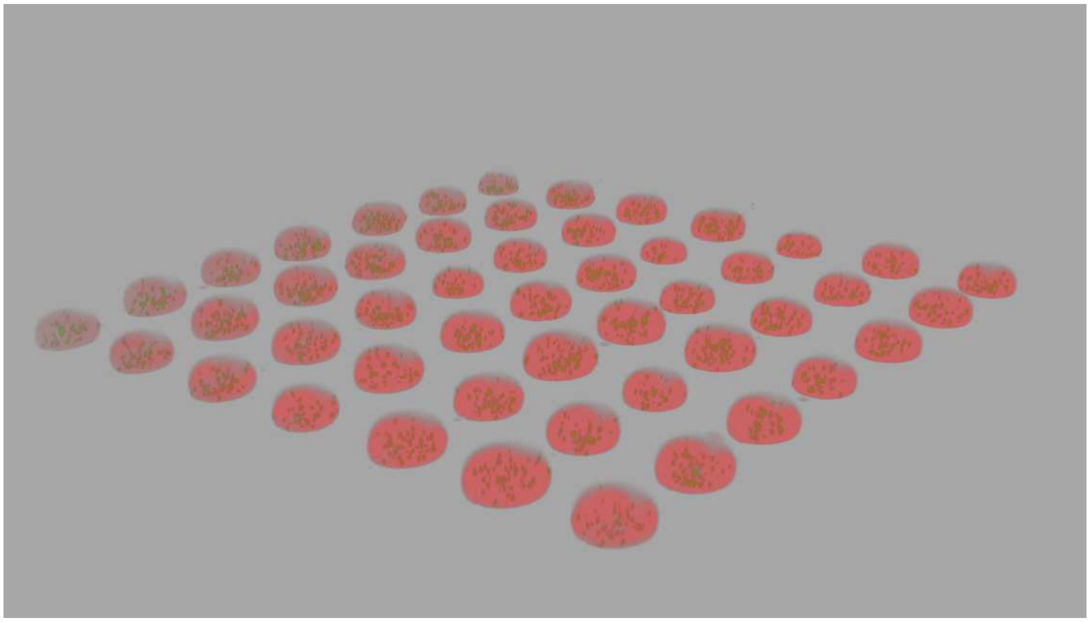

Supplementary Figure 13. 3D confocal microscopy image showing acoustically trapped array of PDDA/ATP coacervate micro-droplets (red) containing sequestered FITC-SiO_2 microparticles (2.5 µm, small green dots). Sequestration of silica microparticles into the acoustically trapped droplets was undertaken by injecting 100 µL of premixed silica microparticles (2.5 µm, 2.5 x 10^{-2} wt.%) and aqueous ATP (100 µL, 50 mM) into a PDDA solution (1 mL, 5 mM monomer; PDDA monomer : ATP = 1, pH =7) contained within the device and subjected to two orthogonal acoustic standing waves (6.76/6.78 MHz, 10 V). The mixtures were stirred to ensure homogeneous formation of the coacervate droplets in the square chamber. 3D confocal microscopy construction of the silica microparticle-containing PDDA/ATP droplets was undertaken by adding sulforhodamine B (10 µL, 1 mM) to the coacervate micro-droplet array after 45 min of acoustic processing.

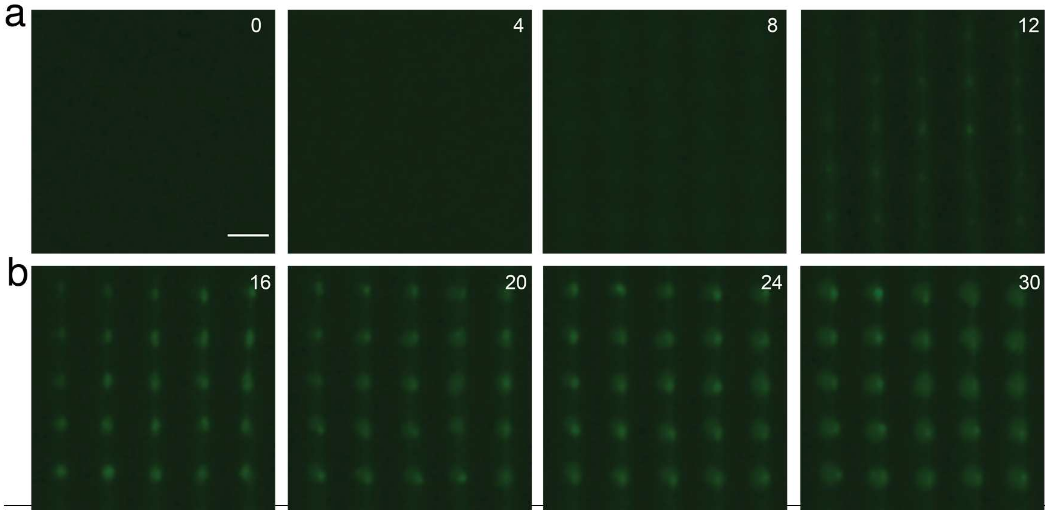

Supplementary Figure 14. (*a,b*) Time-dependent fluorescence microscopy images of acoustically trapped FITC-GOx-containing coacervate micro-droplets recorded at 0, 4, 8, 12 (row (*a*)), and 16, 20, 24 and 30 min (row (*b*)) after addition of ATP (100 µL, 50 mM) into 1 mL of PDDA (5 mM monomer, 8.5 kDa) containing FITC-GOx (1 µg mL^{-1}) in the presence of two orthogonal acoustic standing waves (6.76/6.78 MHz, 10 V; PDDA monomer : ATP = 1, pH = 7). A square pattern of green fluorescence droplets progressively emerged from a green background within 12 min; scale bar = 100 µm.

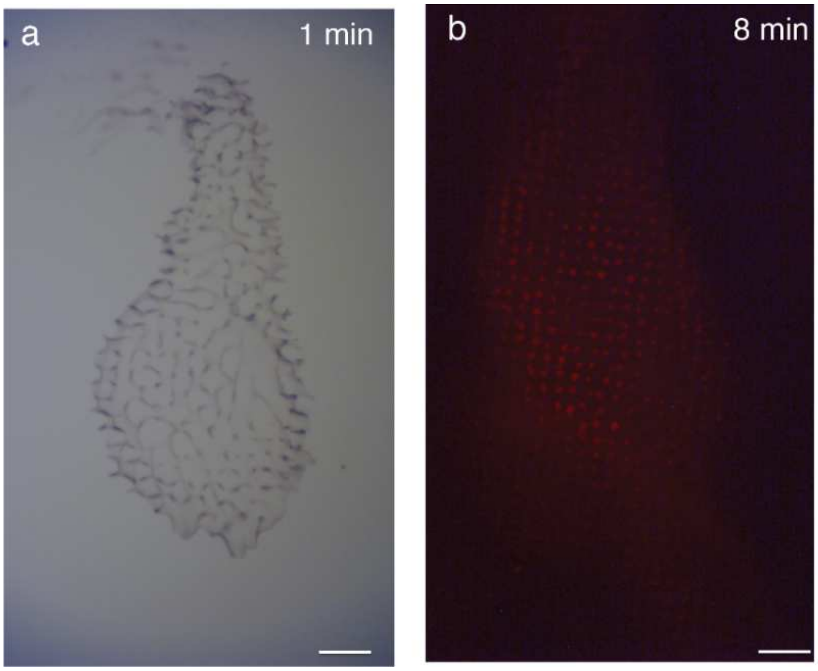

Supplementary Figure 15. Optical (*a*) and corresponding fluorescence (*b*) microscopy images of HRP-containing PDDA/ATP droplets prepared by injection of a PDDA/RITC-HRP (1 µL, 25 mM PDDA, monomer; 0.1 mg mL^{-1} HRP) mixture into an unstirred ATP solution (1 mL, 2.5 mM) under two orthogonal acoustic standing waves (6.76/6.78 MHz, 10 V). After injection, small droplets were formed *in situ* around the location where the PDDA was added, and gradually migrated to the nodes of the acoustic waves to generate a square pattern with spacing of *ca*. 110 µm ($\lambda/2$). The fluorescence microscopy image clearly reveals that RITC-HRP is successfully sequestered in the coacervate droplets. RITC-HRP was excited at λ_{ex} = 515-560 nm with an emission wavelength cut off λ_{em} = 580 nm; scale bars = 400 µm.

Supplementary methods

Materials

All chemicals were used without further purification. Adenosine 5′-triphosphate (ATP), poly(diallyldimethylammonium chloride) (PDDA, 20 wt%, M_w ≈ 100-200 kDa), poly(allylamine hydrochloride) (PAH, M_w ≈ 17.5 kDa), rhodamine isothiocyanate (RITC), fluorescein isothiocyanate (FITC), poly(acrylic acid, sodium salt) (PAA, M_w ≈ 5 kDa), carboxymethyl-dextran sodium salt (CM-D, M_w ≈ 10 - 20 kDa), FITC-tagged CM-D (M_w ≈ 150 kDa), bovine serum albumin (BSA), deoxyribonucleic acid from salmon sperm (DNA, M_w ≈ 1300 kDa), branched polyethylenimine (PEI, M_w ≈ 25 kDa), tris(hydroxymethyl)aminomethane (Tris), glucose oxidase from Aspergillus niger (GOx), peroxidase from horseradish (HRP), amyloglucosidase from Aspergillus niger (AGx), FITC-tagged SiO_2 micro-particles (2.5 μm), FITC-tagged polystyrene nanoparticles (100 nm), *o*-phenylenediamine (*o*-PD), methylene blue, nile red, sulforhodamine B and calcein were purchased from Sigma. 2′,3′-*O*-(2,4,6-trinitrophenyl)adenosine-5′-triphosphate (TNP-ATP, 10 mM, aqueous solution) was obtained from Jena Bioscience. Poly(diallyldimethylammonium chloride) (PDDA, 28 wt%, M_w ≈ 8.5 kDa) was bought from Polysciences Inc. 2-Methoxy(polyethyleneoxy)propyl trimethoxysilane (PEG-TMS) was purchased from ABCR GmbH. DyLight 405 was obtained from Thermo Fisher Scientific Inc. Milli-Q water (18.2 MΩ·cm) was used in the preparation of all aqueous solutions.

Optical and confocal microscopy

Optical microscopy experiments were carried out on a Leica DMI 3000B optical microscope. Fluorescence imaging was performed using a Leica DFC 310FX set up, and dye molecules were excited by using specific filters with the following excitation (λ_{ex}) and emission wavelength cut offs (λ_{em}): DyLight 405, TNP-ATP, 2,3-diaminophenazine, λ_{ex} = 355 - 425 nm and λ_{em} = 455 nm; calcein, FITC, λ_{ex} = 450 - 490 nm and λ_{em} = 510 nm; RITC, sulforhodamine B, nile red, λ_{ex} = 515 - 560 nm and λ_{em} = 580 nm; methylene blue, λ_{ex} = 590 - 650 nm and λ_{em} = 660 nm. All glass slides used for the imaging were functionalized with 2-methoxy(polyethyleneoxy)propyl trimethoxysilane (PEG-TMS).

Confocal microscopy imaging was performed by mounting the custom-made acoustic device on a Leica SP5-II laser scanning microscope attached to a Leica DMI 6000 inverted epifluorescence microscope, and equipped with a ×10 or ×20 objective (0.4 NA and 0.7 NA, respectively). High contrast images against the background solution were obtained after *ca*. 45 minutes of *in situ* pattern formation. 3D reconstructions were processed with Icy software, and all images were consistent with the images shown in the main text. All the glass slides for the imaging were functionalized with PEG-TMS.

Enzyme modifications

Fluorescein-isothiocyanate (FITC) was used to modify glucose oxidase (GOx) and bovine serum albumin (BSA). Rhodamine-isothiocyanate (RITC) was used to modify horseradish peroxidise (HRP). DyLight 405 was used to modify amyloglucosidase (AGx). In general, the

enzymes (10 mL, 4 mg mL^{-1}) were dissolved in sodium carbonate buffered solutions (100 mM, pH = 8), and then a dimethyl sulfoxide (DMSO) solution of FITC (200 µL, 2 mg mL^{-1}), RITC (200 µL, 2 mg mL^{-1}) or DyLight 405 (200 µL, 2 mg mL^{-1}) was added. The reaction mixtures were kept at 4 °C for 12 h, and then dialysed (molecular weight cut 15 kDa) against Milli-Q water over three days with regular water changes. The fluorescently tagged enzymes were then lyophilized and stored in the dark before use in the experiments. Dye concentrations bound to GOx, BSA, HRP AGx were determined by UV/vis spectroscopy ($\varepsilon_{(559\ nm)}$ = 6.21 x 10^4 $M^{-1}cm^{-1}$ for RITC; $\varepsilon_{(485\ nm)}$ = 5.04 x 10^4 M^{-1} cm^{-1} for FITC; $\varepsilon_{(400\ nm)}$ = 3.00 x 10^4 M^{-1} cm^{-1} for DyLight 405), which gave an approximately 1 : 40 ratio of RITC : HRP, 1 : 1 ratio of FITC : GOx, 3 : 1 ratio of FITC : BSA and 1 : 1 ratio DyLight 405: AGx.

Functionalization of PAH with RITC

PAH (10 mL, 10 mg mL^{-1}) was dissolved in 4-(2-hydroxyethyl)-1-piperazinepropanesulfonic acid buffer (EPPS, 100mM, pH = 9.5), and then a DMSO solution of RITC (2.83 mL, 1 mg mL^{-1}) slowly added. The reaction mixture was stirred in the dark for 12 h, and then dialysed (molecular weight cut-off 7 kDa) against Milli-Q water over three days with regular water changes. The, RITC-tagged PAH polymer was lyophilized and stored in the dark before use in the experiments. The dye concentration bound to PAH was determined by UV/vis spectroscopy ($\varepsilon_{(559\ nm)}$ = 6.21 x 10^4 M^{-1} cm^{-1}), which gave an approximately 1 : 10^4 ratio of RITC : PAH-monomer.
